# Diversification of quantitative morphological traits in wheat

**DOI:** 10.1093/aob/mcad202

**Published:** 2024-01-09

**Authors:** Yixiang Shan, Colin P Osborne

**Affiliations:** Plants, Photosynthesis and Soil, School of Biosciences, University of Sheffield, Sheffield S10 2TN, UK; Plants, Photosynthesis and Soil, School of Biosciences, University of Sheffield, Sheffield S10 2TN, UK

**Keywords:** Wheats, domestication, morphology, wheat (*Triticum aestivum* L.), polyploidy, selective breeding, Green Revolution, evolution

## Abstract

**Background and Aims:**

The development and morphology of crop plants have been profoundly altered by evolution under cultivation, initially through unconscious selection, without deliberate foresight, and later by directed breeding. Wild wheats remain an important potential source of variation for modern breeders; however, the sequence and timing of morphological changes during domestication are not fully resolved.

**Methods:**

We grew and measured 142 wheat accessions representing different stages in wheat evolution, including three independent domestication events, and compared their morphological traits to define the morphospace of each group.

**Key Results:**

The results show that wild and domesticated wheats have overlapping morphospaces, but each also occupies a distinct area of morphospace from one another. Polyploid formation in wheat increased leaf biomass and seed weight but had its largest effects on tiller loss. Domestication continued to increase the sizes of wheat leaves and seeds and made wheat grow taller, with more erect architecture. Associated changes to the biomass of domesticated wheats generated more grains and achieved higher yields. Landrace improvement subsequently decreased the numbers of tillers and spikes, to focus resource allocation to the main stem, accompanied by a thicker main stem and larger flag leaves. During the Green Revolution, wheat height was reduced to increase the harvest index and therefore yield. Modern wheats also have more erect leaves and larger flower biomass proportions than landraces.

**Conclusions:**

Quantitative trait history in wheat differs by trait. Some trait values show progressive changes in the same direction (e.g. leaf size, grain weight), whereas others change in a punctuated way at particular stages (e.g. canopy architecture), and other trait values switch directions during wheat evolution (e.g. plant height, flower biomass proportion). Agronomically valued domestication traits arose during different stages of wheat history, such that modern wheats are the product of >10 000 years of morphological evolution.

## INTRODUCTION

Wheat (*Triticum aestivum* L.) is one of the major crops of the world, grown over a land area greater than any other crop (Milla and Osborne, 2019) and accounting for 20 % of food calories globally (Erenstein *et al.*, 2022). The earliest evidence of wheat domestication comes from Neolithic archaeological sites in the western Fertile Crescent (Brown *et al.*, 2009). This ancient history makes wheat one of the oldest crops, and it was one of the species underpinning the first agricultural economies (Abbo and Gopher, 2017) and later grain states (Zhao *et al.*, 2023*a*) in the Middle East. Both the genotype and the phenotype of wheat have changed under domestication and subsequent evolution under selective breeding. Numerous studies have compared wild wheats with domesticated forms, finding a syndrome of traits associated with domestication, including non-brittle rachis, larger seeds and leaves (Evans, 1993), delayed flowering time (Cockram *et al.*, 2007), loss of dormancy (Harlan *et al.*, 1973), greater above-ground biomass (Roucou *et al.*, 2018) and faster growth (Gómez-Fernández *et al.*, 2022).

Many authors consider domestication to be a slow process, occurring across a broad geographical area, with domesticated forms first arising at low frequencies among cultivated stands of wild plants (Tanno and Willcox, 2006). In addition, several domestication traits are complex, presumably controlled by multiple loci, and arise gradually during wheat evolution. Examples of such quantitative traits include plant height (Peng *et al.*, 2003), tillering capacity (Peng *et al.*, 2011) and leaf size (Milla and Matesanz, 2017). All show marked differences in comparisons between wild and domesticated forms. However, there is considerable diversity among accessions and species, and the picture is complicated by changes in ploidy during wheat evolution that are classically associated with gigantism (Fuller, 2007). Therefore, the extent to which quantitative morphological changes have arisen in wheat from polyploidy, domestication and selective breeding remains unclear (Li *et al.*, 2014; Gui *et al.*, 2021).

The diversity of modern wheat is well characterized and provides a useful means to address these questions. Polyploidy, domestication and selective breeding happened at different historical time points and their effects can be inferred via comparisons of extant wheat species. The wild wheats *Triticum urartu* (AA) and *Triticum boeoticum* (AA) are modern representatives of the earliest diploid wheats (Johnson and Dhaliwal, 1976). The first polyploidization event happened 300 000–500 000 years ago, when the wild wheat *Triticum urartu* (AA) formed a natural hybrid with *Aegilops* (*Aegilops speltoides*, genome SS), the closest relative of *Triticum* (Abbo *et al.*, 2014). This hybridization created the wild progenitor of emmer wheat, with the AABB genotype, named *Triticum dicoccoides* (Supplementary Data Fig. S1). Another wild relative, the tetraploid *Triticum araraticum*, probably arose from an independent hybridization of *T. urartu* with *Aegilops* (Supplementary Data Fig. S1) and has the AAGG genome (Badaeva *et al.*, 2022).

People started to cultivate these wild wheats in the Fertile Crescent ~10 000 years ago (Tanno and Willcox, 2006; Faris, 2014). From this time point, there were three independent domestication trajectories (Supplementary Data Fig. S1), each characterized by the loss of natural dispersal via selection for a tough rachis: (1) wild *T. boeoticum* was domesticated to *Triticum monococcum* (einkorn, genome AmAm) (Heun *et al.*, 1997); (2) wild *T. araraticum* was domesticated to *Triticum timopheevii* (Oliveira *et al.*, 2020); and (3) wild *T. dicoccoides* was domesticated to *Triticum dicoccum* (emmer, genome AABB) (Peleg *et al.*, 2011). Domesticated emmer wheat, *T. dicoccum*, underwent a second natural hybridization with another *Aegilops* species (*Aegilops tauschii*, genome DD) 9000 years ago (Dvorak *et al.*, 2012). This event created hexaploid bread wheat (*T. aestivum*, genome AABBDD; Supplementary Data Fig. S1). Subsequent breeding under cultivation turned tetraploid emmer wheat into a landrace type, *Triticum durum* (genome AABB) (Supplementary Data Fig. S1; Bozzini, 1988). Selection for free-threshing means that *T. durum* and *T. aestivum* both have a low degree of glume tenacity and free-threshing habits, which distinguish them from hulled emmer wheat (Peng *et al.*, 2011). Both *T. aestivum* and *T. durum* were subsequently improved during the Green Revolution (Supplementary Data Fig. S1; Byerlee and Traxler, 1995). Modern representatives of these two species are grown on large commercial scales today, while domesticated landraces of emmer and einkorn continue to be grown only on small scales as heritage varieties.

Here, we aim to determine how morphology has changed quantitatively during wheat evolution and to attribute each change to polyploidy, domestication, landrace improvement or modern breeding through the Green Revolution. We compare a diverse range of wheat accessions in a common environment and make four comparisons (Supplementary Data Fig. S2) to infer: (1) the pre-domestication effects of polyploidy across two independent events (*T. urartu vs T. dicoccoides* and *T. urartu vs T. araraticum*); (2) domestication across three independent events (*T. boeoticum vs T.monoccum*, *T. araraticum vs T. timopheevii* and *T. dicoccoides vs T. dicoccum*), evolution of landraces after domestication (*T. dicoccum* vs landraces of *T. durum* or *T. aestivum*), and the Green Revolution (domesticated *T. aestivum* vs modern *T. aestivum*, domesticated *T. durum* vs modern *T. durum*). The novelty of this analysis comes from multiple independent comparisons (Supplementary Data Fig. S2), which sample a diversity of accessions. Our work shows that the pattern of variation in quantitative traits across the four stages differs by trait. Some trait values show progressive changes in the same direction (e.g. leaf size, shoot diameter), some change in a punctuated way at particular stages (e.g. leaf angle), and other trait values show changes in direction during wheat evolution (e.g. plant height, number of tillers).

## MATERIALS AND METHODS

### Plant material

**Fig. 1.**
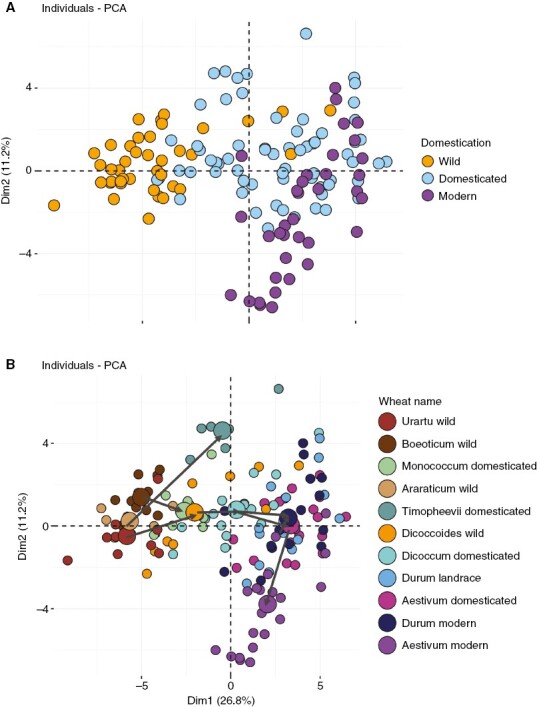
The morphospace occupied by wild and domesticated wheats, presented as a principal components analysis (PCA) for morphological traits during vegetative and reproductive phases. Smaller points correspond to individual plants, while larger points represent species means. (A) The colour coding distinguishes wild from domesticated and modern wheats. (B) The colour coding shows species, as indicated in the key. The black routes track the histories of three domesticated wheat lineages.

We collected many accessions of wheat and cultivated them to measure their morphological characteristics. Sampling of the accessions was structured according to biological status and phylogeny. We first included the wild wheats, both diploid (*T. urartu* and *T. boeoticum*) and tetraploid (*T. dicoccoïdes* and *T. araraticum*) (Fig. 1). For domesticated landraces, we included diploid einkorn (*T. monococcum*), the tetraploid wheats (*T. timopheevii*, *T. dicoccum* and *T. durum*) and domesticated bread wheats (*T. aestivum*). For modern wheats from breeding programmes spanning the Green Revolution, we included durum (*T. durum*) and bread (*T. aestivum*) varieties. In total, we therefore included 11 wheat species in this experiment, representing the diversity of wild and domesticated forms (Supplementary Data Fig. S1; Table S1).

Within this diversity, domesticated bread wheat landraces (*T. aestivum*) were provided by Dr Andrea Harper at the University of York. These originate from Asia, Europe, South America, North America, Africa and Oceania. Many of these landrace wheat lines were gained from the Watkins Collection of the John Innes Centre (Wingen *et al.*, 2014). Modern bread wheats were collected from the National Institute of Agricultural Botany and were parents of the MAGIC Diverse population, a representative collection of UK varieties from 1920–1990 spanning the Green Revolution, which contribute to UK breeding programmes (Gardner *et al.*, 2016). The others were obtained from the Leibniz Institute of Plant Genetics and Crop Plant Research Genebank (Gatersleben, Germany) and the US National Plant Germplasm System (NPGS). In order to sample the diversity for each of these wheat species, we obtained multiple accessions from the recognized wild progenitors and cultivated varieties, including the variation in geographical source, life history (spring or winter) and seed cover (hulled or free-threshing). In total, we had 142 wheat accessions in our experiment, listed in Supplementary Data Table S1. In the following analysis, we combine them according to their scientific name and domestication status. For example, wild *T. aratu* is referred to as ‘urartu wild’. A full list of these abbreviations is provided in Supplementary Data Table S1.

### Growth conditions

For each accession, up to ten seeds were selected randomly and put into the refrigerator at 4 °C for 24 h. After that, the outer glumes were removed, and the seeds were weighed to obtain their mass. Each accession of fresh seeds was germinated in a closed Petri dish, with a wet filter paper put on the bottom (Adamski *et al.*, 2018) and kept in the following conditions in an incubator (versatile environmental test chamber, Panasonic, UK): 12 h dark–12 h light, 20 °C, photosynthetic photon flux density 300 μmol m^−2^ s^−1^ and 60 % relative humidity. Germination took different lengths of time in each accession and was recorded to the nearest day.

Germinated seeds were transplanted (one plant per pot) into trays (4 × 6 cells) containing high-nutrient compost (M3, Levington Horticulture Ltd, Ipswich, UK), supplemented with perlite (Sinclair Nursery Stock Propagation, Levington Horticulture Ltd, Ipswich, UK) in a 3:1 ratio. These pots were labelled and moved into a new controlled-environment growth cabinet (Conviron BDW 40, Conviron, Winnipeg, Manitoba, Canada). This controlled environment, designed for vernalizing winter wheats, was: 12 h dark–12 h light, 4 °C, photosynthetic photon flux density 300 μmol m^−2^ s^−1^ and 60 % relative humidity. Spring wheats were treated in the same way, despite not requiring vernalization, to enable fair comparison of traits with the winter varieties. The vernalization lasted for 6 weeks. During the first week of May 2021, the wheat seedlings were transplanted into pots (15 cm × 15 cm × 20 cm, 3.5 L, LBS Horticulture, UK), with the same soil compost as mentioned above, and moved outdoors into an unshaded area of the Arthur Willis Environment Centre at the University of Sheffield, UK. For each wheat accession, we grew three individual plants, organized randomly and spaced in 5 × 5 plant blocks with 0.25 m distance between plants. In addition to rainwater inputs, the plants were watered as required to keep the soil wet.

### Trait measurements

During wheat growth, we selected and measured some morphological traits that are recognized to influence yield (Supplementary Data Table S2). Among them, dry biomass used one replicate plant for each accession, the final harvest measurement used another, and the third plant was a spare in case one of the others died. Other non-destructive trait measurements were taken in all three repeated samples and used to calculate an average for each accession. All the traits and their shorthand names are listed in Supplementary Data Table S2.

In addition, we used the measured traits to make predictions of yield, harvest index and the area of individual leaves. Expected yield (*Y*) was calculated using the grain weight on one spike (WGS_harvest_) and number of spikelets on one spike (NST_harvest_) at harvest, and the number of spikelets in July (NST_July_), flower biomass in July (BF_July_) and one spike biomass in July (OBS_July_), as follows:


$$\begin{array}{l} Y=\;\;\;\frac{WGS_{harvest}}{NST_{harvest}}\times NST_{July}\times \frac{BF_{July}}{\begin{array}{l} OBS_{July}\; \end{array}}. \end{array}$$ (1)

Final yield (*Y*_f_), was calculated using the NST_harvest_ and NST_July_:


$$\begin{array}{l} Y_{f}=Y\;\times \frac{NST_{harvest}}{NST_{July}}. \end{array}$$ (2)

The harvest index (HI) was calculated using leaf biomass (*B*_L_), flower biomass (*B*_F_) and shoot biomass (*B*_S_) in July:


$$\begin{array}{l} HI=\;\frac{Y_{f}}{B_{L}+B_{F}+B_{S}}. \end{array}$$ (3)

Expected leaf area (LA) followed Schrader *et al.* (2021) and was calculated using leaf length (LL_July_) and leaf width (WL_July_) in July:


$$\begin{array}{l} LA=LL_{July}\times WL_{July}\times 0.75. \end{array}$$ (4)

Tiller loss proportion (LT) was calculated with tiller number in June (NT_June_), spike number in July (NS_July_) and spike number at harvest (NS_harvest_):


$$\begin{array}{lll} LT= & \;\left[maximum\left(NT_{June},\;NS_{July}\right)-\;NS_{harvest}\right]\; & /maximum\;\;\;\left(NT_{June},\;NS_{July}\right). \end{array}$$ (5)

In the calculations of HI, *Y* and *Y*_f_, we removed samples (*n* = 3) in which HI was >0.75, which were regarded as biologically implausible.

### Statistical analysis

Replication in our experiment was at the level of wheat species, such that we could make comparisons among species, accounting for the diversity of accessions within each, but did not compare individual accessions. In doing this, we recognize that landrace and wild accessions are assemblies of seeds collected from a single geographical location, and are not completely uniform for phenotypic traits. In addition, genetic drift for morphological traits might have occurred in material from genebanks, where individuals from an accession are selfed to create ‘pure’ seed stocks.

Data analysis was conducted using Microsoft Office, Excel (https://products.office.com/en-gb/get-started-with-office-2019) and R v.4.0.2 (https://www.r-project.org/). Variation within the dataset of morphological trait values was first described using principal components analysis (PCA), after scaling each trait to standardized values (mean = 0 and s.d. = 1). We used the ‘FactoMineR’ package in R to run the PCA and visualize the resulting morphospace of wild and domesticated groups, then the ‘vegan’ package in R was used for the analysis. We fitted an Envfit model using the ‘rda’ function to test whether biological status or polyploidy consistently influenced wheat morphologies.

To make the multiple planned comparisons outlined in Supplementary Data Fig. S2, we also applied mixed-effects models using the ‘lme4’ packages in R. We selected some of the traits that made high contributions to major axes in the PCA and avoided repeating the analyses for strongly correlated traits. We used the four events described in Table 1 as fixed factors and used wheat species as random effects to run the mixed-effects models. Subsequent ANOVAs on models were done with the ‘lme4Test’ package in R. For domestication and Green Revolution comparisons, we also added the block as a random effect. When applying some of the traits as response variables, the model either failed to converge or converged to a parameter estimate at the boundary of parameter space. In these cases, we removed ‘species’ as a random effect (only in domestication and Green Revolution cases). Finally, we applied a *t*-test to compare traits of wild *T. urartu* and modern *T. aestivum*, the results of which is used as the ultimate contrast between the most ancient species and the present wheat. We also applied Tukey’s HSD test to make pairwise comparisons among wheat species using the ‘agricolae’ package in R.

**Table 1.** Summary of changes in traits during wheat evolution. The orange shaded boxes indicate significant increases, whereas the purple shaded boxes show significant decreases in trait values for the contrast indicated. The column ‘overall’ refers to the comparison of wild *Triticum urartu* and modern *Triticum aestivum*.

Category	Traits	Polyploidization	Domestication	Landrace improvement	Modern breeding	Overall
Architectural traits	Initial plant height	**–**	**–**	**–**	**↓**	**↑**
	Final plant height	**–**	**↑**	**–**	**↓**	**↓**
	Stem diameter	**–**	**–**	**↑**	**–**	**↑**
	Leaf insertion angle	**–**	**↓**	**–**	**↓**	**↓**
Tillering strength	Maximum number of tillers	**–**	**–**	**↓**	**–**	**↓**
	Final number of spikes	**–**	**–**	**↓**	**–**	**↓**
	Proportion of tillers lost	**↑**	**–**	**–**	**–**	**↑**
Biomass allocation	Above-ground biomass	**–**	**–**	**–**	**–**	**↑**
	Shoot biomass	**–**	**–**	**–**	**–**	**↑**
	Leaf biomass	**↑**	**–**	**–**	**–**	**↑**
	Flower biomass	**–**	**–**	**–**	**–**	**↑**
	Flower biomass proportion	**–**	**↓**	**–**	**↑**	**–**
Leaf traits	Leaf length	**–**	**↑**	**–**	**↓**	**↑**
	Leaf width	**–**	**–**	**–**	**–**	**↑**
	Flag leaf length	**–**	**–**	**↑**	**–**	**↑**
	Flag leaf width	**–**	**↑**	**↑**	**–**	**↑**
	One leaf biomass	**↑**	**↑**	**–**	**–**	**↑**
	Expected leaf area	**–**	**–**	**↑**	**–**	**↑**
Yield-related traits	One spike length	**–**	**–**	**–**	**–**	**↑**
	One spike biomass	**–**	**–**	**–**	**–**	**↑**
	Number of spikelets per spike	**–**	**↑**	**–**	**–**	**↑**
	Number of grains per spike	**–**	**↑**	**–**	**↑**	**↑**
	Grain weight per spike	**–**	**↑**	**–**	**↑**	**↑**
	Individual grain weight	**↑**	**↑**	**–**	**–**	**↑**
	Expected yield	**–**	**↑**	**–**	**–**	**↑**
	Harvest index	**–**	**↑**	**–**	**–**	**↑**

## RESULTS

### Morphological variation

Given that morphological traits are likely to be correlated, we began by using PCA to produce a morphospace showing the main axes of variation and important groupings of traits. The morphospace occupied by wild, domesticated and modern wheat species is distinct but overlapping. The species occupy a broad arc across the first two principal component axes (Fig. 1A), such that the morphospace of wild forms overlaps with domesticated forms and that of domesticated forms overlaps with modern wheats. However, there is no morphological overlap between wild and modern wheats. The main effect of domestication has been to increase values of dimension 1 in the PCA (Fig. 1A), which corresponds to greater size of plants, stems and leaves during the vegetative phase of development (Supplementary Data Fig. S1). Alongside this, there is a diversification of low values in dimension 2 (Fig. 1A), which corresponds to shorter height at maturity (Supplementary Data Fig. S1). Modern selective breeding has acted primarily to lower and diversify values of dimension 2 (Fig. 1A), to produce low-stature varieties (Supplementary Data Fig. S1). In broad terms, the results therefore confirm the known effects of domestication in producing gigantism and the effects of modern breeding in shortening plants at maturity. Within these broad patterns there are important differences among species. Polyploidy has had only modest effects on the sizes of plants and their organs in both *T. araraticum* and *T. dicoccoides* (dimension 1, Fig. 1B). However, the enlargement of plants during domestication is greater in the tetraploids (*T. dicoccum* and *T. timopheevi*) than in the diploid (*T. monococcum*) (dimension 1, Fig. 1B). Conversely, the final increase in height associated with domestication is largely observed in *T. timopheevi*, with only limited or no height gains in *T. dicoccum* (emmer) and *T. monococcum* (einkorn) (dimension 2, Fig. 1B). The breeding of landraces from *T. dicoccum* has had more uniform effects in both *T. aestivum* and *T. durum*, with both showing increases in size compared with *T. dicoccum* during the vegetative phase (dimension 1, Fig. 1B), but no reduction in final height (dimension 2, Fig. 1B). Finally, Green Revolution breeding has had limited effects on size during the vegetative phase (dimension 1, Fig. 1B), with a focus on shorter final height in *T. aestivum* but not *T. durum* (dimension 2, Fig. 1B).

### Architectural traits

**Fig. 2.**
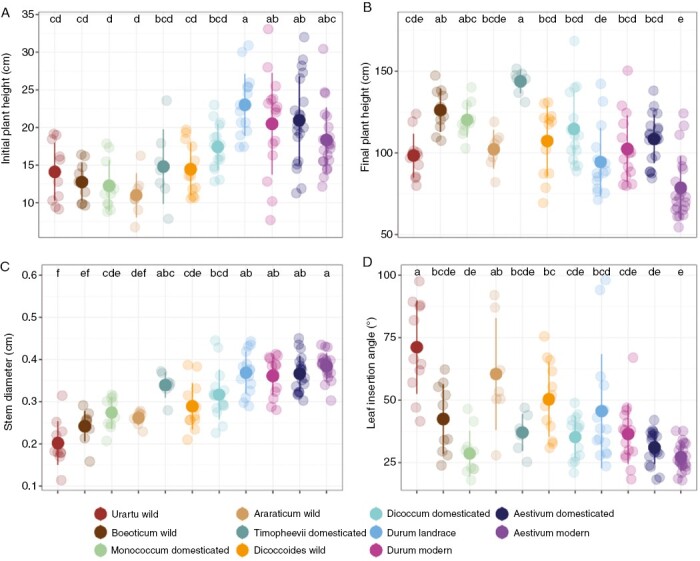
Diversity in morphology and architecture among wheat species. (A) Initial plant height. (B) Plant height at the end of vegetative growth. (C) Main stem diameter at the end of vegetative growth. (D) Leaf insertion angle on the main stem. Different letters above points indicate significant differences at *P* < 0.05 using Tukey’s multiple comparison test.

Wheat diversification after domestication has been associated with progressive increases in height and stem diameter during the vegetative phase, such that there is ≤10-fold variation in height among wild and domesticated forms during May (Fig. 2A). In contrast, although plant height at maturity varies >3-fold after wheat diversification, the most prominent effects are associated with the short-stemmed modern cultivars of bread wheat released after the Green Revolution and the tall stature of *T. timopheevii* noted earlier (Fig. 2B). Moreover, based on data from previous studies (*Scott *et al.*, 2021*) of modern varieties in our experiments, we found variation before and after the Green Revolution. In Supplementary Data Fig. S3A, we analysed the relationship between the release date for each variety and plant height and found that the height of modern *T. aestivum* decreased progressively in newer varieties. This explains the effects of the Green Revolution and the several data points with higher values within modern *T. aestivum* in Fig. 2B. However, we did not find obvious relationships between height and the release date in modern *T. durum*. Within the overall trends, substantial variation within groups means that finer-grained details are harder to resolve. Polyploidy has no effects on plant height in wild wheat species, such that there is no evidence of wild tetraploid wheats being taller than wild diploid species during the vegetative phase (Fig. 2A; Table 1) or at maturity (Fig. 2B; Table 1). Stem diameter was greater in *T. dicoccoides* than in *T. uratu*, but the equivalent comparison for *T. araraticum* vs. *T. uratu* was not significant (Fig. 2C; Table 1). Height and stem diameter were also generally unaffected across the three independent domestication events (Fig. 2A, B; Table 1), with the exception of height at maturity and stem diameter in *T. araraticum* vs *T. timopheevii*, where the domesticated form is significantly taller and with a thicker stem than the wild species (Fig. 2B; Table 1). Landrace diversification has resulted in taller forms of both durum and bread wheats, but the overall effect is significant only during the vegetative phase in durum (Fig. 2A, B; Table 1). However, stems are thicker in both cases (Fig. 2C; Table 1). Finally, as expected, modern breeding has typically shortened the height at maturity for bread wheat in comparison to its landraces (Fig. 2B; Table 1). This decreased height was observed only in *T. aestivum* and not in *T. durum* in our experiment.

Leaf insertion angle has also shown a progressive decrease during wheat evolution, to produce modern forms with much more erect, compact leaf canopies compared with the lax, spreading canopies of the wild ones (Fig. 2D; Table 1). In consequence, there is no overlap in values between wild *T. uratu* and modern *T. aestivum* (Fig. 2D). The largest changes are observed across the three independent domestication events, and the difference between *T. aestivum* landraces and modern cultivars is not statistically significant (Fig. 2D).

### Tillering strength

**Fig. 3.**
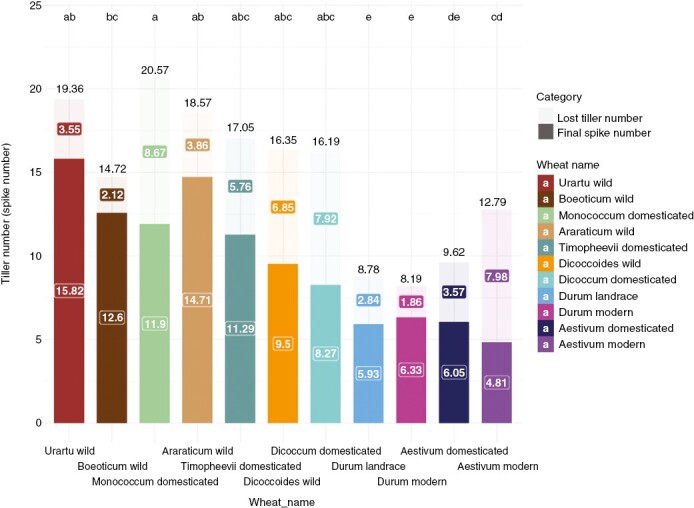
Diversity in the number of tillers and spikes among wheat species. For each species, the solid colour shows the final number of spikes at maturity, and the paler colour shows the maximum number of tillers that we observed during development. The difference between these values gives tiller loss, highlighted in the coloured boxes. The numbers correspond to trait values. Different letters above points indicate significant differences at *P* < 0.05 using Tukey’s multiple comparison test on the maximum number of tillers.

Wild wheats tend to have strong tillering to occupy space and increase their reproductive potential. Polyploid formation has exacerbated spike loss such that larger proportions of tillers do not produce spikes (Table 1). Unexpectedly, we found no evidence that domestication had consistent impacts of tillering across the three domestication events (Table 1). However, the tillering strength of einkorn (*T. boeoticum* vs *T. monococcum*) increased after domestication (Fig. 3), although subsequent tiller loss meant that the final number of spikes of *T. monococcum* is not higher than that of its wild relatives. A reduced number of spikes at harvest after landrace improvement has arisen through a different mechanism. Selective breeding during landrace diversification has limited the final number of spikes by decreasing the maximum number of tillers, without a change in the proportion of tillers that are lost without setting seeds (Fig. 3; Table 1). We found no evidence of further changes in tillering arising from modern breeding programmes. Overall, therefore, improved modern polyploid wheats produce fewer tillers and lose a greater proportion than wild diploid wheats, but these changes did not occur during either domestication or modern breeding.

### Biomass allocation

**Fig. 4.**
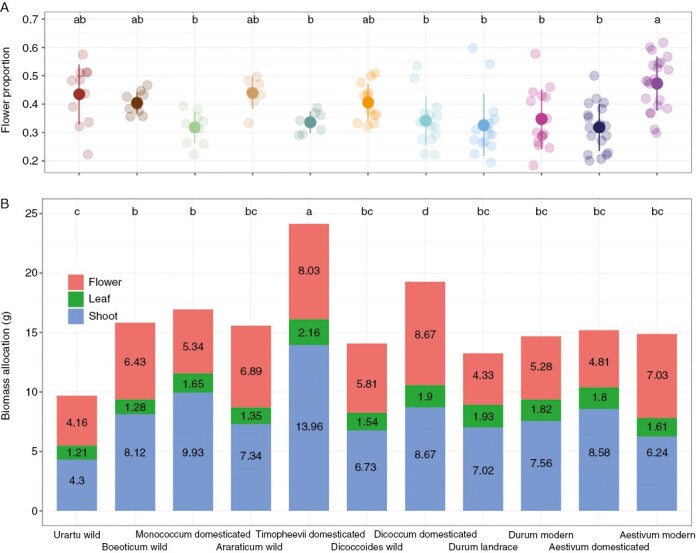
Diversity in flower proportion and biomass allocation at anthesis among wheat species. (A) Flower biomass relative to the whole above-ground biomass. (B) Biomass allocation to the flower, leaf and shoot (stem and leaf sheaths). The numbers show the biomass values for each tissue. Different letters above points indicate significant differences at *P* < 0.05 using Tukey’s multiple comparison test.

There was no overall difference in above-ground vegetative biomass between the wild and modern varieties in our pot experiment, potentially reflecting the equal access to soil nutrients that each plant had available. However, we found evidence that the allocation of biomass between flowers, shoots and leaves at anthesis has changed during wheat evolution. Unexpectedly, domestication across three independent events has not brought an obvious increase in total flower biomass. Instead, the selective breeding of modern bread wheat varieties during the Green Revolution is largely responsible for the greater flower biomass of modern wheats in comparison to wild wheats, and its proportion relative to above-ground biomass (Fig. 4A; Table 1). We analysed modern wheat flower proportions further and found a significant effect of variety release date (Supplementary Data Fig. S3B). As the date gets closer to the present, the flower biomass proportion increases, showing the expected directed increase in harvest index associated with the Green Revolution and modern breeding programmes. This phenomenon was found only in *T. aestivum* and was not significant in *T. durum*. Conversely, domestication across three independent events has decreased relative allocation of biomass to flowering (Table 1). At the same time, leaf biomass increased across these domestication events, continuing a pattern that started across the polyploidy events in wild wheats (Fig. 4B; Table 1). However, there have been no further changes during landrace diversification and modern breeding, and overall leaf biomass does not differ between wild and modern varieties (Fig. 4B; Table 1). Domesticated wheats tend to have larger above-ground biomass than their wild relatives, although there are no statistically significant differences (Fig. 4B). Wheat has the largest above-ground biomass in domesticated *T. timopheevii*, which has larger shoot and leaf biomass than its wild progenitor, *T. araraticum* (Fig. 4B; Table 1). Meanwhile, *T. timopheevii* is also larger than the other domesticated wheats, *T. dicoccum* and *T. monococcum*.

### Leaf traits

**Fig. 5.**
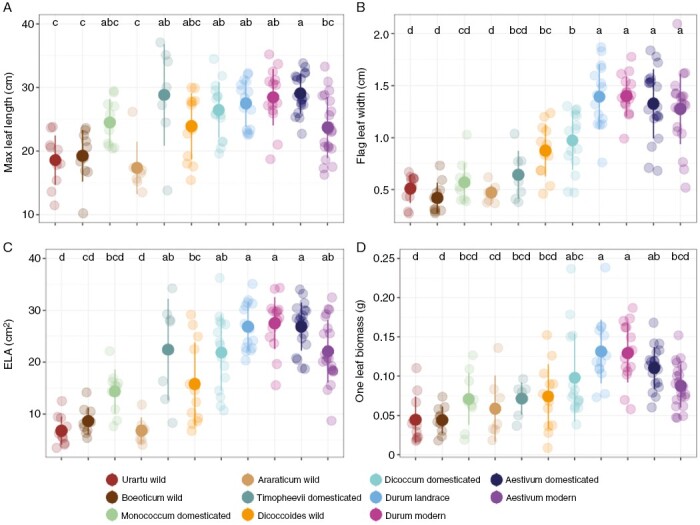
Diversity of leaf traits among wheat species. (A) Maximum leaf length. (B) Flag leaf width. (C) Individual leaf area (ELA), which is calculated using the maximum leaf length and width. (D) Individual leaf biomass. Different letters above points indicate significant differences at *P* < 0.05 using Tukey’s multiple comparison test.

Wheat evolution under cultivation has altered leaf traits substantially. In particular, maximum leaf length and flag leaf width were substantially increased during domestication (Table 1). Fig. 5A shows that leaf length increased most notably during the domestication of *T. araraticum* to *T. timopheevii*. Likewise, the width of the flag leaf was significantly increased during landrace improvement (Fig. 5B; Table 1), enlarging individual leaves (Table 1). Although our analysis did not reveal a significant increase in leaf area during domestication (Table 1), the species comparison for the independently domesticated *T. araraticum* vs *T. timopheevii* showed a strong increase (Fig. 5C). Mixed models found that individual leaf biomass increased continuously throughout both polyploid formation and domestication (Table 1), but the HSD test did not find significant differences among neighbouring species representing the sequence from wild to modern wheats (Fig. 5D). Overall, leaf size showed a consistently increasing trend throughout the diversification of wheat, with the exception of modern varieties, which had shorter leaf lengths than *T. aestivum* landraces (Fig. 5A; Table 1). However, modern polyploid wheat leaves still have a much larger area than those of their ancient diploid progenitor, *T. urartu*.

### Yield-related traits

**Fig. 6.**
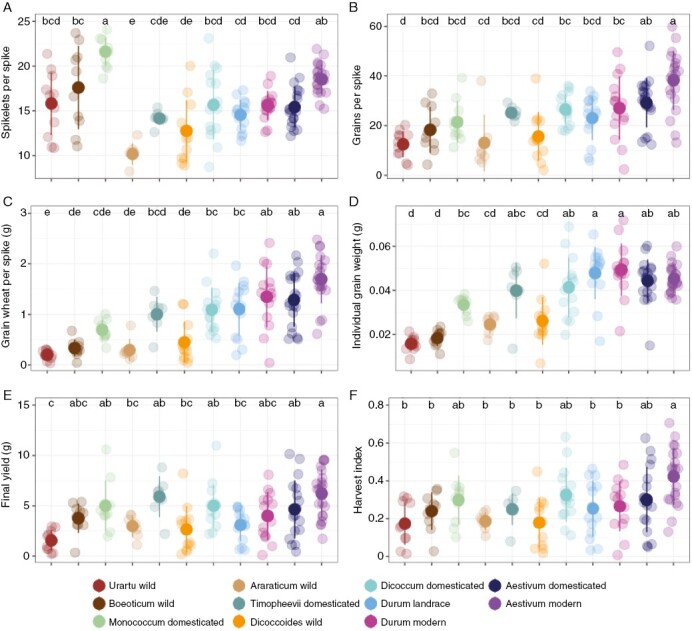
Diversity of yield-related traits among wheat species. (A) Number of spikelets on the largest spike. (B) Number of grains on the largest spike. (C) Weight of grain on the largest spike. (D) Mean individual grain weight. (E) Expected final yield for one plant, considering tiller loss. (F) Expected harvest index. Different letters above points indicate significant differences at *P* < 0.05 using Tukey’s multiple comparison test.

Yield-related traits are of greatest concern from agronomic and economic perspectives. During domestication, the number of spikelets increased significantly. Fig. 6A shows large differences among wild and domesticated forms in einkorn (*T. boeoticum* vs *T. monococcum*) and emmer (*T. araraticum* vs *T. timopheevii* and *T. dicoccoides* vs *T. dicoccum*) comparisons. The number of grains and grain weight also show an overall increasing trend throughout wheat diversification (Fig. 6B, C). The analysis of wheat species shows that this increase is slow (Fig. 6B, C), and the huge gap between modern *T. aestivum* and wild *T. urartu* is formed gradually. However, mixed-effects models point to two stages when changes are particularly pronounced, domestication and the Green Revolution (Table 1). The number of grains per spike and the mass of individual grains have both increased, with a consequent increase in the total grain mass per spike (Fig. 6B–D; Table 1). However, changes are not obvious at other stages (Fig. 6A–C; Table 1). Polyploid formation increases the individual grain weight significantly (Table 1), but in the contrasts among wheat species, the effects of polyploidy and landrace improvement are relatively small (Fig. 6D). Domestication and selective breeding have brought higher yields in wheat, as expected. However, our experiment indicates that improvements have not been continuous, with the major change in yield being associated with domestication, as evidenced across three independent events (Table 1). In contrast, neither polyploidy in wild plants nor landrace improvement and modern breeding have had effects of an equivalent magnitude to those of domestication (Fig. 6E; Table 1) in the conditions used in our study (individual plants grown in pots). The anticipated increase in harvest index associated with short-stature plants after the Green Revolution is apparent in our data, but is not statistically significant owing to substantial variation in this emergent trait within landraces and modern varieties of bread wheat (Fig. 6F; Table 1). In contrast, the statistical power associated with three domestication events shows statistically significant increases of harvest index in these cases (Fig. 6F; Table 1).

## DISCUSSION

In this study, we compared the morphological traits of wheat and investigated the stages of evolution at which they occurred. Our findings revealed that morphological changes during the evolution of wheat have been episodic, with different evolutionary trajectories for each trait. During each period, historical events caused wheat to improve its strategies for adapting to the external environment or to meet the artificial requirements of farmers.

### Distinct patterns of phenotypic variation through history

The phenotypic variation observed in wheat reflects its growth strategies across the four examined periods (i.e. polyploid formation, domestication, landrace improvement and the Green Revolution). The main priority for wild wheats is to reproduce and survive. Chromosome doubling increases the genome sizes of wheats (Özkan *et al.*, 2010), causing leaf size and seed size to increase. Our study is consistent with previous work, showing that tetraploid wheat (AABB and AAGG) has thicker leaves, with more dry matter and chlorophyll per unit area than diploid ones (Kaminski *et al.*, 1990), suggesting that polyploidization promotes wheat photosynthesis as a source of increased vigour. The seed sizes of polyploids are typically larger than those of their diploid relatives (Dhawan and Lavania, 1996), and larger seeds provide competitive advantages in crop progenitors (Preece *et al.*, 2017). Compared with diploids, larger tetraploid seeds often result in greater growth vigour, as seen in muskmelons (Batra, 1952) and subterranean clover (Hutton and Peak, 1954). Larger seed and leaf biomass as characteristics of gigantism are considered typical features of polyploidy (Heslop-Harrison *et al.*, 2023), although neither is found with statistical significance in our study. This might be attributable to the slow growth speed of polyploids during the adult stage that has been observed previously (Bose and Choudhury, 1962). Further work supports this interpretation by comparing growth in diplod–tetraploid pairs of *Phlox drummondii*, finding that tetraploids tended to produce lower intrinsic rates of leaf growth and fewer but larger flowers (Garbutt and Bazzaz, 1983). This finding might explain the increase in tiller loss we observed in polyploid wheat, although the numbers of tillers and spikes did not show significant variation. Therefore, we can infer that, although polyploidy influences early size and vigour, leaf size and tiller retention, it does not have obvious overall effects on growth.

**Fig. 7.**
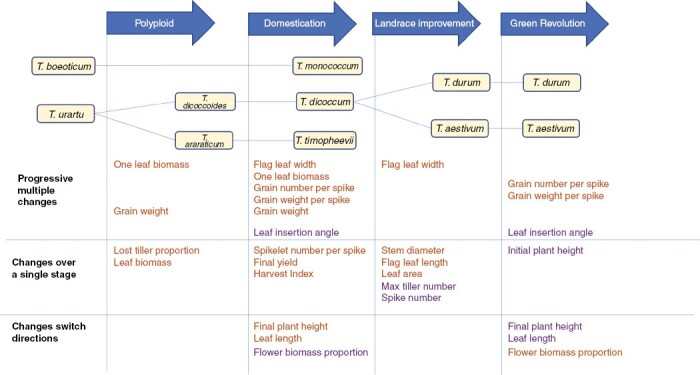
Conceptual diagram of wheat evolution, showing trait changes at four evolutionary stages (polyploid formation, domestication, landrace improvement and the Green Revolution). The traits coloured orange have increased values, whereas those coloured purple have decreased values.

Increased seed and leaf sizes continued through the process of domestication. In fact, the larger size of seeds might have a strong positive relationship with larger leaves (Hodgson *et al.*, 2017), and they have been a crucial factor in species selection for cultivation (Preece *et al.*, 2015). During domestication, seed size is thought to have increased through selection on plant size and production or via natural selection for competitive ability, which indirectly selected for larger sizes of individual plant parts (Jones *et al.*, 2021). The nature of selection during domestication is controversial. In ancient cultivation, increased seed size might come from unconscious natural selection (Harlan *et al.*, 1973) by farmers because they lacked foresight of the potential for selective breeding (Kluyver *et al.*, 2017). In this case, the collection of plants from the wild or their cultivation in farming environments drove natural selection for traits that adapted crops for the new environment or harvest system (Zohary, 2004). However, Darwin believed that farmers were unconsciously selecting large seeds as a domestication trait by planting larger seeds each generation and discarding smaller ones (Darwin, 1859). This led to changes in the population without any deliberate planning (Darwin, 1868). Most recently, Jones *et al.* (2021) argued that domesticated traits might be selected for by plant competition in anthropogenic environments. Our study cannot distinguish between these mechanisms, but we did find that various traits, including leaf size, plant height and grain mass, all showed an increase consistent with previous ideas of domesticated plant morphology as gigantism (Fig. 7; Milla and Matesanz, 2017; Gómez-Fernández *et al.*, 2022).

Our findings of a decrease in biomass allocation to flowers with domestication, despite the associated increase in yield, is at first sight a contradiction. However, the result is consistent with previous work for emmer and einkorn wheat that showed reduced allocation to chaff (non-seed reproductive biomass) linked to domestication (Preece *et al*., 2017). Thus, seeds are favoured in domesticated wheats at the expense of other flowering structures.

Landrace improvement in wheat has led to reduced tillering and the promotion of main stem growth. The numbers of both tillers and spikes decrease but, at the same time, flag leaf size and stem diameter increase (Fig. 7). These changes reflect a classic trade-off between the number of spikes and grain weight (Xie and Sparkes, 2021). In a field situation, fewer spikes per plant lead to higher yields, because decreased numbers of spikes can be compensated by high planting density (Li *et al.*, 2016). Previous work in rice has also suggested that decreased numbers of spikes would lead to sufficient grain filling and high starch content (Panigrahi *et al.*, 2019). However, our work does not find greater grain weight in landraces compared with less improved domesticated forms.

Our data for Green Revolution varieties show the well-known trade-off between investment in the stem and grains, seen as reduced plant height and improved yield. This variation has been observed in many studies (e.g. Mann, 1997; Hedden, 2003 ; Würschum *et al.*, 2017). Both initial and final plant height are decreased, while investment in grain is promoted via increased flower proportion, number and weight of grains (Fig. 7). Moreover, leaf size and insertion angle decrease further, meaning that intensive breeding has limited neighbour competition to favour investment in grains.

### Continuity and opposition of trait changes

Some trait values showed equivalent changes across multiple stages (Fig. 7). For example, leaf size increased during both polyploidy–domestication and domestication–landrace transitions. However, in wild plants the maximum leaf biomass increased, whereas in landrace improvement the flag leaf size increased. This might be because the flag leaf is more relevant to ear development (Sanchez-Bragado *et al.*, 2014) and is preferred by farmers or breeders. Domestication and the Green Revolution both increased grain weight per spike and the number of grains per spike, which are more directly relevant to yield. Moreover, the leaf insertion angle decreased at both these stages. Leaf insertion angle, as one of the factors influencing wheat above-ground architecture, was thought to have changed during polyploidization (Li *et al.*, 2014). However, our work provides a wider range of wheat species at each ploidy and domestication level and suggests that leaf insertion has been most influenced by the two farming stages. The increased density of farmed plants might have selected for more erect architectures, a conclusion supported by recent genetic evidence (Zhao *et al.*, 2023*b*).

However, there are some other traits showing opposing changes between domestication and the Green Revolution, indicating that modern breeding has, in some respects, needed to undo the effects of domestication. For example, both plant height and leaf length are important in early wheat improvement, but their reduction through selective breeding has improved flower biomass allocation. Thus, evolution during domestication led to trait combinations that are undesirable in modern agriculture. For example, selection for larger leaves and increased height helped to acquire above-ground resources (light and space) in early cultivated environments. However, these effects of gigantism in crops were detrimental for yields from the crop population as a whole. Crop plants need to cooperate, rather than compete, to maximize population yield (Anten and Vermeulen, 2016), such that crops with intermediate individual fitness have the highest yield per unit area (Weiner *et al.*, 2017).

### Future directions in phenotyping work and how phenotype benefits yield

Owing to time and cost limitations, we were unable to study wheat root phenotypes, which might play an important role in wheat evolution. For example, domestication increases biomass allocation to the shoot instead of the root (Qin *et al.*, 2012), and the Green Revolution decreased root biomass further in elite wheat varieties (Waines and Ehdaie, 2007). As fertilizer applications increased under cultivation, wheats needed to allocate fewer resources to roots to acquire water and nitrogen (Gioia *et al.*, 2015). We would therefore expect the individual competitiveness of modern wheat to decrease below ground.

Phenotyping of diverse wheat accessions has high current relevance, owing to the focus on traits from wild progenitors in modern breeding programmes (Skovmand *et al.*, 2001; Leigh *et al.*, 2022). Wheat germplasm diversity is generally thought to have decreased through artificial selection (Reif *et al.*, 2005; Haudry *et al.*, 2007; Kilian *et al.*, 2010). However, our work has shown that in some respects this loss has been associated with the diversification of trait values. The wild morphospace does not cover the domesticated one completely, because new trait values were generated during wheat evolution. Crop diversification compensates for domestication bottlenecks by capturing part of the genetic diversity of its progenitors and by generating new diversity at a relatively fast pace (Dubcovsky and Dvorak, 2007). Thus, domesticated and modern morphospaces expand beyond that of the wild species, which represents valuable trait diversity available to breeders.

### Conclusions

In conclusion, our study shows that wheat phenotypic evolution is a long and complex process. Some traits have been changed continuously in the same directions through crop history, whereas other traits have changed in opposite directions during two or more periods. Differences between wild and modern wheats are therefore the product of multiple phases of historical change, in which natural and artificial selection have been important in various ways. This long history of crop diversification has generated valuable traits for use in modern breeding work. Understanding the trajectory of wheat phenotypic evolution can therefore promote agricultural and germplasm improvement.

## SUPPLEMENTARY DATA

Supplementary data are available at *Annals of Botany* online and consist of the following.

Fig. S1: wheat evolutionary history and relationships. Fig. S2: four evolutionary events considered and the statistical model for each. Fig. S3: trait variation of modern wheat in relation to acquisition year. Table S1: wheat accessions used in this experiment. Table S2: traits measured and their abbreviations.

mcad202_suppl_Supplementary_Figures_S1-S3_Tables_S1-S2
