# Supplementary material for: Diversification of quantitative morphological traits in wheat
Source: Ann Bot. 2024 Jan 9;133(3):413–26. doi: 10.1093/aob/mcad202 (PMC11006538; doi:10.1093/aob/mcad202)
Supplement: mcad202_suppl_Supplementary_Figures_S1-S3_Tables_S1-S2 [file mcad202_suppl_supplementary_figures_s1-s3_tables_s1-s2.docx]

**Supporting Information**

**Article title:** Diversification of quantitative morphological traits in wheat

**Authors:** Yixiang Shan and Colin Osborne

The following Supporting Information is available for this article:

Fig. S1. Wheat evolutionary history and relationships.

Fig. S2. Four evolutionary events considered and the statistical model for each.

Fig. S3. Trait variation of modern wheat in relation to acquisition year.

Table S1. Wheat accessions used in this experiment.

Table S2. Traits measured and their abbreviations.


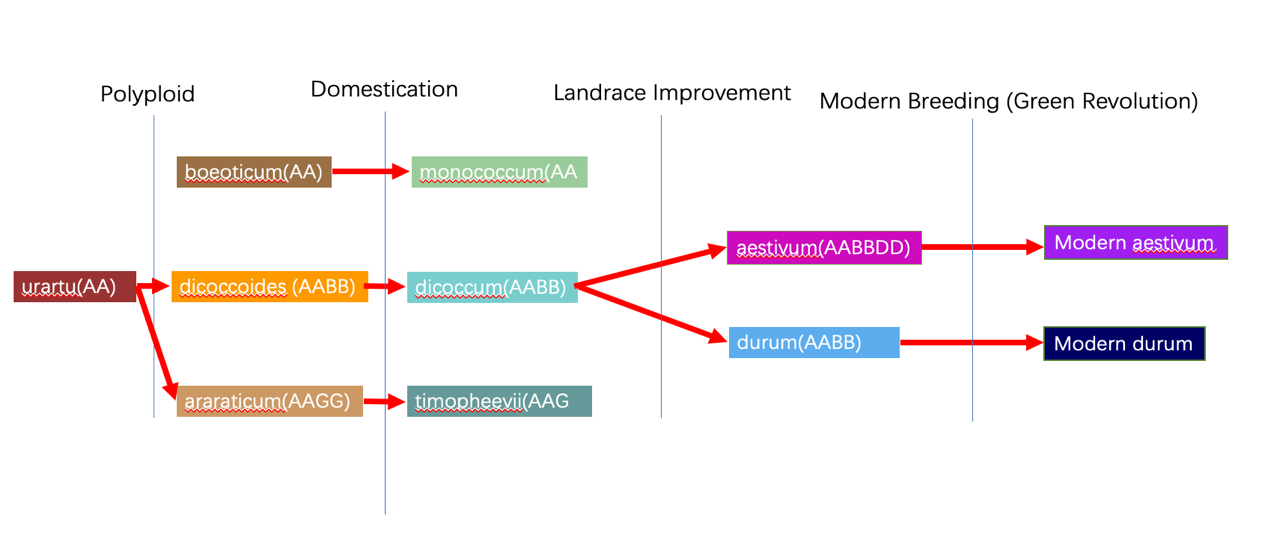


Figure S1: Wheat evolutionary history and relationships.

Figure S2: Four evolutionary events considered and the statistical model for each. The statistical method was a mixed effects model, which is specified in each case. The four events are regarded as fixed factors in the models, while wheat species are random effects.


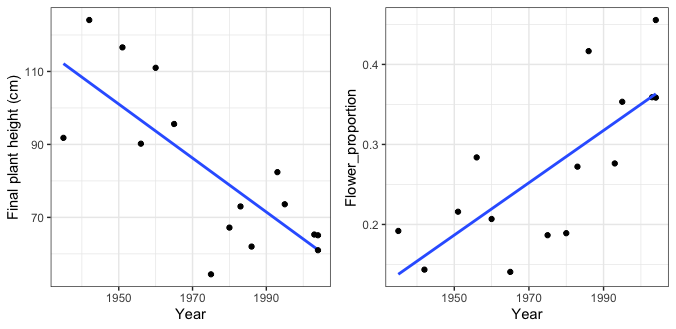


Figure S3: Trait variation of modern wheat in relation to acquisition year. (A) Final plant height. Fitting a linear model with an ANOVA yielded a p-value of 0.0006964, which means the year has a highly significant effect on the final plant height; (B) Proportion of flower biomass to total aboveground biomass of plants. Fitting a linear model with an ANOVA yielded a p-value of 0.001164, which means the year has a highly significant effect on biomass allocation.

Table S1: Wheat accessions used in this experiment.

| **Wheat accession** | **Domestication** | **Genome** | **Abbreviation** | **Species name** | **Code** | **Source** |
| --- | --- | --- | --- | --- | --- | --- |
| York 102+aestivum25 | domesticated | hexaploid | aestivum domesticated | aestivum | York 102 | University of York |
| York 198+aestivum27 | domesticated | hexaploid | aestivum domesticated | aestivum | York 198 | University of York |
| York 209+aestivum4 | domesticated | hexaploid | aestivum domesticated | aestivum | York 209 | University of York |
| York 229+aestivum29 | domesticated | hexaploid | aestivum domesticated | aestivum | York 229 | University of York |
| York 239+aestivum17 | domesticated | hexaploid | aestivum domesticated | aestivum | York 239 | University of York |
| York 246+aestivum28 | domesticated | hexaploid | aestivum domesticated | aestivum | York 246 | University of York |
| York 271+aestivum5 | domesticated | hexaploid | aestivum domesticated | aestivum | York 271 | University of York |
| York 286+aestivum16 | domesticated | hexaploid | aestivum domesticated | aestivum | York 286 | University of York |
| York 297+aestivum30 | domesticated | hexaploid | aestivum domesticated | aestivum | York 297 | University of York |
| York 299+aestivum6 | domesticated | hexaploid | aestivum domesticated | aestivum | York 299 | University of York |
| York 302+aestivum11 | domesticated | hexaploid | aestivum domesticated | aestivum | York 302 | University of York |
| York 310+aestivum13 | domesticated | hexaploid | aestivum domesticated | aestivum | York 310 | University of York |
| York 311+aestivum7 | domesticated | hexaploid | aestivum domesticated | aestivum | York 311 | University of York |
| York 312+aestivum12 | domesticated | hexaploid | aestivum domesticated | aestivum | York 312 | University of York |
| York 321+aestivum15 | domesticated | hexaploid | aestivum domesticated | aestivum | York 321 | University of York |
| York 328+aestivum10 | domesticated | hexaploid | aestivum domesticated | aestivum | York 328 | University of York |
| York 334+aestivum9 | domesticated | hexaploid | aestivum domesticated | aestivum | York 334 | University of York |
| York 335+aestivum31 | domesticated | hexaploid | aestivum domesticated | aestivum | York 335 | University of York |
| York 75+aestivum23 | domesticated | hexaploid | aestivum domesticated | aestivum | York 75 | University of York |
| TRI 10324+dicoccum4 | domesticated | tetraploid | dicoccum domesticated | dicoccum | TRI 10324 | IPK |
| TRI 13158+dicoccum15 | domesticated | tetraploid | dicoccum domesticated | dicoccum | TRI 13158 | IPK |
| TRI 14077+dicoccum5 | domesticated | tetraploid | dicoccum domesticated | dicoccum | TRI 14077 | IPK |
| TRI 14734+dicoccum13 | domesticated | tetraploid | dicoccum domesticated | dicoccum | TRI 14734 | IPK |
| TRI 16880+dicoccum7 | domesticated | tetraploid | dicoccum domesticated | dicoccum | TRI 16880 | IPK |
| TRI 17038+dicoccum6 | domesticated | tetraploid | dicoccum domesticated | dicoccum | TRI 17038 | IPK |
| TRI 17634+dicoccum8 | domesticated | tetraploid | dicoccum domesticated | dicoccum | TRI 17634 | IPK |
| TRI 19232+dicoccum14 | domesticated | tetraploid | dicoccum domesticated | dicoccum | TRI 19232 | IPK |
| TRI 19294+dicoccum16 | domesticated | tetraploid | dicoccum domesticated | dicoccum | TRI 19294 | IPK |
| TRI 2215+dicoccum9 | domesticated | tetraploid | dicoccum domesticated | dicoccum | TRI 2215 | IPK |
| TRI 28049+dicoccum17 | domesticated | tetraploid | dicoccum domesticated | dicoccum | TRI 28049 | IPK |
| TRI 2884+dicoccum12 | domesticated | tetraploid | dicoccum domesticated | dicoccum | TRI 2884 | IPK |
| TRI 29820+dicoccum11 | domesticated | tetraploid | dicoccum domesticated | dicoccum | TRI 29820 | IPK |
| TRI 6141+dicoccum10 | domesticated | tetraploid | dicoccum domesticated | dicoccum | TRI 6141 | IPK |
| TRI 9542+dicoccum3 | domesticated | tetraploid | dicoccum domesticated | dicoccum | TRI 9542 | IPK |
| CItr 14712+durum3 | domesticated | tetraploid | durum landrace | durum | CItr 14712 | IPK |
| CItr 14978+durum4 | domesticated | tetraploid | durum landrace | durum | CItr 14978 | IPK |
| CItr 15024+durum5 | domesticated | tetraploid | durum landrace | durum | CItr 15024 | IPK |
| CItr 5083+durum1 | domesticated | tetraploid | durum landrace | durum | CItr 5083 | IPK |
| TRI 10513+durum15 | domesticated | tetraploid | durum landrace | durum | TRI 10513 | IPK |
| TRI 14570+durum22 | domesticated | tetraploid | durum landrace | durum | TRI 14570 | IPK |
| TRI 14690+durum16 | domesticated | tetraploid | durum landrace | durum | TRI 14690 | IPK |
| TRI 1542+durum9 | domesticated | tetraploid | durum landrace | durum | TRI 1542 | IPK |
| TRI 26410+durum20 | domesticated | tetraploid | durum landrace | durum | TRI 26410 | IPK |
| TRI 26511+durum8 | domesticated | tetraploid | durum landrace | durum | TRI 26511 | IPK |
| TRI 2721+durum12 | domesticated | tetraploid | durum landrace | durum | TRI 2721 | IPK |
| TRI 2928+durum13 | domesticated | tetraploid | durum landrace | durum | TRI 2928 | IPK |
| TRI 29588+durum19 | domesticated | tetraploid | durum landrace | durum | TRI 29588 | IPK |
| TRI 5508+durum14 | domesticated | tetraploid | durum landrace | durum | TRI 5508 | IPK |
| W 2604+durum24 | domesticated | tetraploid | durum landrace | durum | W 2604 | IPK |
| PI 418583+monococcum8 | domesticated | diploid | monococcum domesticated | monococcum | PI 418583 | NPGS |
| PI 428159+monococcum9 | domesticated | diploid | monococcum domesticated | monococcum | PI 428159 | NPGS |
| TRI 19406+monococcum10 | domesticated | diploid | monococcum domesticated | monococcum | TRI 19406 | IPK |
| TRI 28132+monococcum15 | domesticated | diploid | monococcum domesticated | monococcum | TRI 28132 | IPK |
| TRI 28139+monococcum11 | domesticated | diploid | monococcum domesticated | monococcum | TRI 28139 | IPK |
| TRI 28142+monococcum17 | domesticated | diploid | monococcum domesticated | monococcum | TRI 28142 | IPK |
| TRI 28145+monococcum14 | domesticated | diploid | monococcum domesticated | monococcum | TRI 28145 | IPK |
| TRI 28175+monococcum13 | domesticated | diploid | monococcum domesticated | monococcum | TRI 28175 | IPK |
| TRI 28176+monococcum12 | domesticated | diploid | monococcum domesticated | monococcum | TRI 28176 | IPK |
| TRI 28186+monococcum16 | domesticated | diploid | monococcum domesticated | monococcum | TRI 28186 | IPK |
| CItr 15205+timopheevii1 | domesticated | diploid | timopheevii domesticated | timopheevii | CItr 15205 | IPK |
| PI 119442+timopheevii2 | domesticated | diploid | timopheevii domesticated | timopheevii | PI 119442 | NPGS |
| PI 221421+timopheevii9 | domesticated | diploid | timopheevii domesticated | timopheevii | PI 221421 | NPGS |
| PI 272530+timopheevii4 | domesticated | diploid | timopheevii domesticated | timopheevii | PI 272530 | NPGS |
| PI 282932+timopheevii5 | domesticated | diploid | timopheevii domesticated | timopheevii | PI 282932 | NPGS |
| PI 352512+timopheevii6 | domesticated | diploid | timopheevii domesticated | timopheevii | PI 352512 | NPGS |
| TRI 4349+timopheevii8 | domesticated | diploid | timopheevii domesticated | timopheevii | TRI 4349 | IPK |
| ALCHEMY14 | modern | hexaploid | aestivum modern | modern_wheat | ALCHEMY | NIAB |
| BANCO3 | modern | hexaploid | aestivum modern | modern_wheat | BANCO | NIAB |
| BERSEE18 | modern | hexaploid | aestivum modern | modern_wheat | BERSEE | NIAB |
| BROMPTON19 | modern | hexaploid | aestivum modern | modern_wheat | BROMPTON | NIAB |
| CLAIRE22 | modern | hexaploid | aestivum modern | modern_wheat | CLAIRE | NIAB |
| COPAIN2 | modern | hexaploid | aestivum modern | modern_wheat | COPAIN | NIAB |
| CORDIALE7 | modern | hexaploid | aestivum modern | modern_wheat | CORDIALE | NIAB |
| FLAMINGO11 | modern | hexaploid | aestivum modern | modern_wheat | FLAMINGO | NIAB |
| GLADIATOR15 | modern | hexaploid | aestivum modern | modern_wheat | GLADIATOR | NIAB |
| HEREWARD6 | modern | hexaploid | aestivum modern | modern_wheat | HEREWARD | NIAB |
| HOLDFAST10 | modern | hexaploid | aestivum modern | modern_wheat | HOLDFAST | NIAB |
| KLOKA20 | modern | hexaploid | aestivum modern | modern_wheat | KLOKA | NIAB |
| MARIS FUNDIN4 | modern | hexaploid | aestivum modern | modern_wheat | MARIS FUNDIN | NIAB |
| RIALTO12 | modern | hexaploid | aestivum modern | modern_wheat | RIALTO | NIAB |
| ROBIGUS9 | modern | hexaploid | aestivum modern | modern_wheat | ROBIGUS | NIAB |
| SLEPNER8 | modern | hexaploid | aestivum modern | modern_wheat | SLEPNER | NIAB |
| SOISSONS1 | modern | hexaploid | aestivum modern | modern_wheat | SOISSONS | NIAB |
| SPARK21 | modern | hexaploid | aestivum modern | modern_wheat | SPARK | NIAB |
| STEADFAST16 | modern | hexaploid | aestivum modern | modern_wheat | STEADFAST | NIAB |
| STETSON17 | modern | hexaploid | aestivum modern | modern_wheat | STETSON | NIAB |
| XI 19/13 | modern | hexaploid | aestivum modern | modern_wheat | XI 19/13 | NIAB |
| TRI 10271+durum23 | modern | tetraploid | durum modern | durum | TRI 10271 | IPK |
| TRI 16641+durum27 | modern | tetraploid | durum modern | durum | TRI 16641 | IPK |
| TRI 19047+durum28 | modern | tetraploid | durum modern | durum | TRI 19047 | IPK |
| TRI 19241+durum39 | modern | tetraploid | durum modern | durum | TRI 19241 | IPK |
| TRI 26968+durum41 | modern | tetraploid | durum modern | durum | TRI 26968 | IPK |
| TRI 2930+durum33 | modern | tetraploid | durum modern | durum | TRI 2930 | IPK |
| TRI 3216+durum30 | modern | tetraploid | durum modern | durum | TRI 3216 | IPK |
| TRI 3615+durum31 | modern | tetraploid | durum modern | durum | TRI 3615 | IPK |
| TRI 6328+durum29 | modern | tetraploid | durum modern | durum | TRI 6328 | IPK |
| TRI 6998+durum25 | modern | tetraploid | durum modern | durum | TRI 6998 | IPK |
| TRI 7662+durum38 | modern | tetraploid | durum modern | durum | TRI 7662 | IPK |
| TRI 880+durum26 | modern | tetraploid | durum modern | durum | TRI 880 | IPK |
| TRI 9776+durum36 | modern | tetraploid | durum modern | durum | TRI 9776 | IPK |
| TRI 9836+durum42 | modern | tetraploid | durum modern | durum | TRI 9836 | IPK |
| TRI 9936+durum34 | modern | tetraploid | durum modern | durum | TRI 9936 | IPK |
| PI 361859+araraticum1 | wild | tetraploid | araraticum wild | araraticum | PI 361859 | NPGS |
| PI 427998+araraticum2 | wild | tetraploid | araraticum wild | araraticum | PI 427998 | NPGS |
| TRI 11345+araraticum6 | wild | tetraploid | araraticum wild | araraticum | TRI 11345 | IPK |
| TRI 11354+araraticum4 | wild | tetraploid | araraticum wild | araraticum | TRI 11354 | IPK |
| TRI 18515+araraticum3 | wild | tetraploid | araraticum wild | araraticum | TRI 18515 | IPK |
| TRI 18534+araraticum5 | wild | tetraploid | araraticum wild | araraticum | TRI 18534 | IPK |
| TRI 7388+araraticum8 | wild | tetraploid | araraticum wild | araraticum | TRI 7388 | IPK |
| PI 352276+boeoticum2 | wild | diploid | boeoticum wild | boeoticum | PI 352276 | NPGS |
| PI 352503+boeoticum3 | wild | diploid | boeoticum wild | boeoticum | PI 352503 | NPGS |
| PI 352505+boeoticum4 | wild | diploid | boeoticum wild | boeoticum | PI 352505 | NPGS |
| PI 355522+boeoticum5 | wild | diploid | boeoticum wild | boeoticum | PI 355522 | NPGS |
| PI 407640+boeoticum7 | wild | diploid | boeoticum wild | boeoticum | PI 407640 | NPGS |
| PI 418580+boeoticum8 | wild | diploid | boeoticum wild | boeoticum | PI 418580 | NPGS |
| PI 427447+boeoticum9 | wild | diploid | boeoticum wild | boeoticum | PI 427447 | NPGS |
| PI 427465+boeoticum10 | wild | diploid | boeoticum wild | boeoticum | PI 427465 | NPGS |
| PI 427466+boeoticum11 | wild | diploid | boeoticum wild | boeoticum | PI 427466 | NPGS |
| PI 427637+boeoticum12 | wild | diploid | boeoticum wild | boeoticum | PI 427637 | NPGS |
| PI 256029+dicoccoides1 | wild | tetraploid | dicoccoides wild | dicoccoides | PI 256029 | NPGS |
| PI 266841+dicoccoides2 | wild | tetraploid | dicoccoides wild | dicoccoides | PI 266841 | NPGS |
| PI 352323+dicoccoides4 | wild | tetraploid | dicoccoides wild | dicoccoides | PI 352323 | NPGS |
| PI 352325+dicoccoides5 | wild | tetraploid | dicoccoides wild | dicoccoides | PI 352325 | NPGS |
| PI 352326+dicoccoides6 | wild | tetraploid | dicoccoides wild | dicoccoides | PI 352326 | NPGS |
| PI 362036+dicoccoides7 | wild | tetraploid | dicoccoides wild | dicoccoides | PI 362036 | NPGS |
| PI 428016+dicoccoides8 | wild | tetraploid | dicoccoides wild | dicoccoides | PI 428016 | NPGS |
| TRI 14095+dicoccoides15 | wild | tetraploid | dicoccoides wild | dicoccoides | TRI 14095 | IPK |
| TRI 18505+dicoccoides14 | wild | tetraploid | dicoccoides wild | dicoccoides | TRI 18505 | IPK |
| TRI 18530+dicoccoides10 | wild | tetraploid | dicoccoides wild | dicoccoides | TRI 18530 | IPK |
| TRI 18539+dicoccoides9 | wild | tetraploid | dicoccoides wild | dicoccoides | TRI 18539 | IPK |
| TRI 9865+dicoccoides16 | wild | tetraploid | dicoccoides wild | dicoccoides | TRI 9865 | IPK |
| PI 401411+urartu1 | wild | diploid | urartu wild | urartu | PI 401411 | NPGS |
| PI 427328+urartu2 | wild | diploid | urartu wild | urartu | PI 427328 | NPGS |
| PI 487271+urartu3 | wild | diploid | urartu wild | urartu | PI 487271 | NPGS |
| PI 662225+urartu4 | wild | diploid | urartu wild | urartu | PI 662225 | NPGS |
| TRI 17119+urartu7 | wild | diploid | urartu wild | urartu | TRI 17119 | IPK |
| TRI 17122+urartu8 | wild | diploid | urartu wild | urartu | TRI 17122 | IPK |
| TRI 17128+urartu6 | wild | diploid | urartu wild | urartu | TRI 17128 | IPK |
| TRI 17129+urartu5 | wild | diploid | urartu wild | urartu | TRI 17129 | IPK |
| TRI 17148+urartu11 | wild | diploid | urartu wild | urartu | TRI 17148 | IPK |
| TRI 17161+urartu12 | wild | diploid | urartu wild | urartu | TRI 17161 | IPK |
| TRI 6735+urartu14 | wild | diploid | urartu wild | urartu | TRI 6735 | IPK |

Table S2: Traits measured and their abbreviations. All biomass measurements used dry mass after oven drying.

| Date | Trait | Age ref (days) | Explanation |
| --- | --- | --- | --- |
| Apr-21 | Seed_weight | 0 | individual seed weight, g |
| Apr-21 | Germination_delay | 10 | delay before germination , days |
| May-21 | Plant_height_May | 40 | shown as [Initial plant height]; choose main stem, extend it and measure the length, cm |
| May-21 | Architecture_height_May | 40 | max plant height from soil in natural state, cm |
| May-21 | Leaf_number_May | 40 | leaf number at one plant in May |
| May-21 | Tiller_number_May | 40 | tiller number in May |
| May-21 | Leaf_length_May | 40 | the maximum leaf length in May, cm |
| Jun-21 | Plant_height_June | 70 | plant height in June, cm |
| Jun-21 | Architecture_height_June | 70 | plant height in natural state, cm |
| Jun-21 | Leaf_number_onestem_June | 70 | leaf number on the largest stem |
| Jun-21 | Leaf_length_June | 70 | length of the largest leaf of one plant in June, cm |
| Jun-21 | Leaf_width_June | 70 | width of the largest leaf of one plant in June, cm |
| Jun-21 | Leaf_position_June | 70 | location on the leaf where width is maximal, ratio |
| Jun-21 | Leaf_insertion_June | 70 | shown as [Leaf insertion angle]; insertion angle of leaves, angle to stem |
| Jun-21 | Leaf_curtivation_June | 70 | leaf curvature - angle between the bottom and top of leaf blade, angle |
| Jun-21 | Shoot_hair_June | 70 | if shoot has white hair, Yes = 1, No = 0 |
| Jun-21 | Weed_June | 70 | if the pot has weeds. Yes= 1, No =0 |
| Jun-21 | Tiller_number_June | 70 | branch number in June |
| Jul-21 | Spike_number_July | 100 | spike number in July |
| Jul-21 | Spike_length_July | 100 | length of the largest spike, cm |
| Jul-21 | Node_number_July | 100 | node number on the main stem |
| Jul-21 | Stem_diameter_July | 100 | shown as [Stem diameter]; largest diameter on the main stem, cm |
| Jul-21 | Peduncle_length_July | 100 | peduncle length in July, cm |
| Jul-21 | Plant_height_July | 100 | shown as [Final plant height]; plant height in July, cm |
| Jul-21 | Architecture_height_July | 100 | plant height in natural state, cm |
| Jul-21 | Flag_leaf_length_July | 100 | leaf length of the flag leaf on the main stem, cm |
| Jul-21 | Flag_leaf_width_July | 100 | shown as [Flag leaf width]; leaf width of the flag leaf on the main stem, cm |
| Jul-21 | Awn_length_July | 100 | the longest awn length, cm |
| Jul-21 | One_leaf_length_July | 100 | shown as [Max leaf length]; length of the largest leaf (not include flag leaf) in July, cm |
| Jul-21 | One_leaf_width_July | 100 | width of the largest leaf (not flag leaf) in July, cm |
| Jul-21 | One_leaf_mass_July | 100 | biomass of the largest leaf (not flag leaf) in July, cm |
| Jul-21 | Flower_mass_July | 100 | biomass of all the spikes on one plant, g |
| Jul-21 | Leaf_mass_July | 100 | biomass of all the leaves on one plant, g |
| Jul-21 | Shoot_mass_July | 100 | biomass of all the shoots on one plant, g |
| Jul-21 | Leaf_biomass_July | 100 | biomass of all the leaves on one plant, g |
| Jul-21 | Shoot_biomass_July | 100 | biomass of all the shoots on one plant, g |
| Jul-21 | Flower_biomass_July | 100 | biomass of all the spike on one plant, g |
| Jul-21 | One_leaf_biomass_July | 100 | shown as [One leaf biomass]; biomass of the largest leaf on the main stem, g |
| Jul-21 | Spikelet_number_July | 100 | spikelet number of the largest spike on the main stem |
| Jul-21 | One_spike_biomass_July | 100 | biomass of the largest spike on the main stem, g |
| Jul-21 | Internode_biomass_July | 100 | biomass of the second internode the main stem, g |
| Jul-21 | Peduncle_biomass_July | 100 | biomass of the peduncle on the main stem, g |
| Sep-21 | Spike_number_harvest | 160 | spike number at one plant in harvest (consider spike loss) |
| Sep-21 | Spikelet_number_onespike_harvest | 160 | shown as [Spikelet number at one spike]; spikelet number of the largest spike in harvest |
| Sep-21 | Spike_length_harvest | 160 | length of the largest spike in harvest, cm |
| Sep-21 | Plant_height_harvest | 160 | plant height at harvest, cm |
| Sep-21 | Leaf_cur_angle_harvest | 160 | angle between consecutive leaves along a stem for the lower phytomers, angle |
| Sep-21 | Spike_ratio_harvest | 160 | ratio of the spike number at harvest to the largest tiller number recorded during development |
| Sep-21 | Leafpetiole_ratio_harvest | 160 | fraction of leaf biomass partitioned to the sheath |
| Sep-21 | Flag_leafpetiole_ratio_harvest | 160 | fraction of flag leaf biomass partitioned to the sheath |
| Sep-21 | Grain_number_onespike_harvest | 160 | shown as [Grain number at one spike]; grain number of the largest spike on the main stem |
| Sep-21 | Grain_weight_onespike_harvest | 160 | shown as [Grain weight at one spike]; average yield of the largest spike on the main stem, g |
| Sep-21 | Grain_weight | 160 | Shown as [Individual grain wegiht]; individual grain weight from harvest, g |
